# Activity of Corallopyronin A Against ESKAPEE Pathogens: Potential and Translational Implications

**DOI:** 10.3390/antibiotics15070665

**Published:** 2026-07-08

**Authors:** Jennifer M. Colquhoun, Kara W. Marshall, Miriam Grosse, Birthe Sandargo, Kenneth Pfarr, Andrea Schiefer, Achim Hoerauf, William M. Shafer, Philip N. Rather

**Affiliations:** 1 Research Service, Atlanta VA Medical Center, Decatur, GA 30033, USAwshafer@emory.edu (W.M.S.); prather@emory.edu (P.N.R.); 2 Department of Microbial Drugs, Helmholtz Centre for Infection Research, 38124 Braunschweig, Germany; miriam.grosse@helmholtz-hzi.de (M.G.); birthe.sandargo@helmholtz-hzi.de (B.S.); 3 German Center for Infection Research (DZIF), Partner Site Hannover-Braunschweig, 38124 Braunschweig, Germany; 4 Institute for Medical Microbiology, Immunology and Parasitology, University Hospital Bonn, University of Bonn, 53113 Bonn, Germany; kenneth.pfarr@ukbonn.de (K.P.); andrea.schiefer@uni-bonn.de (A.S.); achim.hoerauf@ukbonn.de (A.H.); 5 German Center for Infection Research (DZIF), Partner Site Bonn-Cologne, 53113 Bonn, Germany; 6 Department of Microbiology and Immunology, Emory University School of Medicine, Atlanta, GA 30307, USA; 7 Emory Antibiotic Resistance Center, Emory University School of Medicine, Atlanta, GA 30307, USA

**Keywords:** *Acinetobacter baumannii*, CorA, Corallopyronin A, RNA polymerase, ESKAPEE, rifampin resistance, biofilm

## Abstract

**Background**: Corallopyronin A (CorA) is a bacterial RNA polymerase inhibitor that binds a site distinct from rifamycins, but its activity across the ESKAPEE pathogen panel and its translational potential remain incompletely defined. **Methods**: CorA activity was evaluated against representative ESKAPEE pathogens using broth microdilution assays ± polymyxin B nonapeptide (PMBN). Activity was benchmarked against rifampin (Rif). Resistance, cross-resistance, serum activity, in vivo efficacy in the *Galleria mellonella* wax moth larva model, and biofilm disruption were assessed. **Results**: CorA inhibited *Acinetobacter baumannii* (minimal inhibitory concentration [MIC] = 16–32 µg/mL), including MDR isolates, with susceptibility enhanced 4–32-fold by efflux disruption or membrane permeabilization. In contrast, most other Gram-negative ESKAPEE pathogens required PMBN for activity, while Gram-positive organisms were intrinsically susceptible. Rif was consistently more potent than CorA across the panel. Rif-resistant *A. baumannii* and *Klebsiella pneumoniae* remained fully susceptible to CorA, confirming the absence of cross-resistance. Fractional inhibitory concentration (FIC) index analysis revealed pharmacological indifference between CorA and Rif, with no synergy or antagonism detected. CorA activity was abolished in 50% serum conditions and was not restored by PMBN, consistent with serum sequestration; no efficacy was observed in a *G. mellonella* infection model at doses up to 20 mg/kg despite Rif demonstrating significant protection. Notably, CorA reduced established *A. baumannii* biofilms by ∼3–4 log colony-forming units per mL (CFU/mL) across concentrations ≥4× MIC after 24 h of treatment. **Conclusions**: CorA exhibits selective activity against *A. baumannii* and retains efficacy against Rif-resistant strains. While high serum binding reduces in vitro activity and efficacy was not observed in the *G. mellonella* model at a lower dose than in other in vivo models, established in vivo activity against other pathogens demonstrates that serum binding does not preclude therapeutic utility. These findings highlight both the translational considerations for CorA against ESKAPEE pathogens and potential niches for its application, including filarial nematodes, biofilm-associated infections and combination strategies.

## 1. Introduction

The ESKAPEE pathogens—*Enterococcus faecium*, *Staphylococcus aureus*, *Klebsiella pneumoniae*, *Acinetobacter baumannii*, *Pseudomonas aeruginosa*, *Enterobacter* species, and *Escherichia coli*—represent the leading causes of healthcare-associated infections worldwide, and their increasing antimicrobial resistance poses a critical threat to public health [1,2]. Among these, *A. baumannii* has been classified as a critical-priority pathogen by the World Health Organization (WHO) due to its extraordinary capacity to acquire and accumulate resistance determinants, such as carbapenemases, extended-spectrum β-lactamases, or efflux pump overexpression, leading to pan-drug-resistant phenotypes [3,4]. Despite the urgent need for new treatment options, the antibiotic development pipeline remains sparse, with most candidate compounds representing derivatives of existing structural scaffolds [5].

Rifampin (Rif) and related rifamycins represent one of the few antibiotic classes that target RNA polymerase (RNAP), the essential enzyme responsible for bacterial transcription. Rifamycins bind within the RNAP β-subunit (RpoB) rifamycin resistance-determining region (RDDR), physically occluding the RNA exit tunnel and thereby blocking transcription elongation [6]. Although Rif has broad-spectrum activity and is a cornerstone of tuberculosis treatment, its clinical utility against ESKAPEE pathogens is limited: it is rarely used as monotherapy due to rapid resistance emergence, and many Gram-negative pathogens are intrinsically less susceptible due to outer membrane permeability barriers. Nevertheless, Rif serves as a useful mechanistic benchmark for evaluating novel RNAP inhibitors, as its established spectrum and resistance mechanisms provide a reference framework for interpreting new compound activity.

Corallopyronin A (CorA) is a natural product and RNAP inhibitor of myxobacterial origin that targets the RNAP switch region, a structural element distinct from the rifamycin binding site [7,8,9,10,11]. This unique mechanism of action confers several theoretical advantages: CorA is not subject to preexisting Rif resistance, it may be used in combination with rifamycins to target RNAP through two independent sites, and its distinct pharmacophore opens structural space for new medicinal chemistry campaigns. CorA has demonstrated activity against several Gram-positive and Gram-negative species, including *S. aureus*, *Neisseria gonorrhoeae*, and the obligate intracellular pathogens *Chlamydia trachomatis*, *Rickettsia typhi* and *Orientia tsutsugamushi* [12,13,14,15,16]. In addition, CorA targets *Wolbachia* endobacteria of filarial nematodes, demonstrating activity against three filarial species (*Litomosoides sigmodontis* in mice and Mongolian gerbils, *Onchocerca ochengi* in mice, and *Dirofilaria immitis* in dogs), and clinical development is anticipated to begin in 2027 [13,15,17,18,19,20,21]. However, systematic evaluation of CorA activity across the full ESKAPEE panel, direct comparison to Rif, and characterization of cross-resistance have not been reported, and while substantial in vivo research has established CorA efficacy against the above pathogens, translational assessment in the context of ESKAPEE priority pathogens specifically remains incomplete.

Here, we present a comprehensive evaluation of CorA activity against *A. baumannii* and representative ESKAPEE pathogens, benchmarked against Rif. We characterize the molecular basis of CorA resistance in *A. baumannii*, assess cross-resistance relationships with Rif, and evaluate CorA activity under physiologically relevant conditions, including serum, an invertebrate infection model, and established biofilms. Together, these studies define both the promise and the translational constraints of CorA as an RNAP-targeting therapeutic candidate.

## 2. Results

### 2.1. Antimicrobial Activity of CorA Against A. baumannii

Corallopyronin A (CorA) has displayed antimicrobial activity against a broad range of bacterial species; however, its efficacy against *A. baumannii* had not previously been characterized. We first tested antimicrobial activity against the reference strain ATCC 17978 and observed an MIC of 16 µg/mL (Table 1). Critically, given that the CorA MIC vs. *S. aureus* was in the ranges previously published [17,22,23], we concluded that the CorA preparation was active. Importantly, increasing outer membrane permeability due to the addition of polymyxin B nonapeptide (PMBN; 50 µg/mL), a permeabilization agent that lacks antimicrobial activity (MIC > 256 µg/mL), or stepwise addition of subinhibitory colistin resulted in a 16–32-fold decrease in MIC. Consistent with observations in *N. gonorrhoeae* [12,24], genetic inactivation of the major efflux pump AdeIJK (Δ*adeJ*) similarly enhanced susceptibility ~4-fold, whereas overexpression of AdeIJK via an *adeN* mutation conferred a 2-fold increase in resistance. Deletion of *adeB*, *adeG::kan*, or *adeL*::kan (leading to AdeFGH overexpression) had no significant effect on CorA susceptibility. These data indicate that AdeIJK-mediated efflux and outer membrane permeability are the primary determinants of intrinsic CorA susceptibility in *A. baumannii*. Notably, to evaluate CorA activity in a clinically relevant context, we included three carbapenem-resistant *A. baumannii* (CRAB) clinical isolates—AB5075, MU1984, and PR322. Testing CorA against these CRAB isolates revealed MICs of 16–32 µg/mL, comparable to ATCC 17978. Each isolate showed enhanced susceptibility upon PMBN treatment or Δ*adeJ* deletion. Importantly, stepwise addition of subinhibitory colistin concentrations further reduced the CorA MIC from 32 µg/mL to 1–4 µg/mL against AB5075 (Table 1).

### 2.2. CorA Targets A. baumannii RpoB

**Figure 1:**
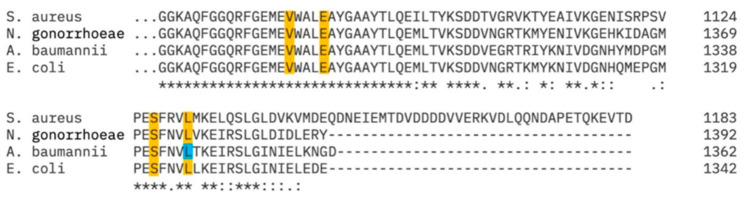
RpoB protein sequence alignment and conservation of amino acids conferring CorA resistance. ClustalW alignment of ~60 amino acids of the C-terminal region of RpoB from *S. aureus* (NCBI #WP_000918667.1), *N. gonorrhoeae* (NCBI #WP_003690105.1), *A. baumannii* (NCBI #AKQ28316.1), and *E. coli* (NCBI #AAC76961.1). Residue highlighted in blue corresponds to *A. baumannii* L1345, which is mutated to phenylalanine (L1345F) to confer CorA resistance. Residues highlighted in orange are known resistance-conferring positions in other bacterial species. An asterisk (*) indicates positions with fully conserved residues across all sequences. Colons (:) denote strongly similar properties, while periods (.) indicate weakly similar, semi-conserved substitutions. Hyphens (-) represent alignment gaps.

To define the mechanism of CorA action in *A. baumannii*, we selected for CorA-resistant mutants in an efflux-deficient, outer membrane-compromised background (Δ*adeB* Δ*adeG* Δ*adeJ* Δ*surA*, known as 17978.EM3) on 4× MIC CorA agar to minimize the recovery of efflux-based resistance. The resistance frequency was ~4.5 × 10^−9^. Whole genome sequencing of four independent resistant mutants identified a leucine-to-phenylalanine substitution at amino acid position 1345 of RpoB (L1345F). Introduction of the *rpoB-L1345F* allele into wildtype ATCC 17978 by allelic exchange conferred a 16-fold increase in CorA MIC alone or in the presence of PMBN (Table 1), confirming RpoB as the molecular target. Protein sequence alignment of RpoB from *S. aureus*, *N. gonorrhoeae*, *A. baumannii*, and *E. coli* demonstrates that the L1345 residue is conserved across these species and corresponds to previously characterized CorA resistance mutations in *N. gonorrhoeae* (L1376) and *S. aureus* (L1131) (Figure 1), indicating a conserved switch region binding mode across bacterial phyla [12,24].

### 2.3. Comparative Activity Across ESKAPEE Pathogens and Rif Benchmarking

To place CorA activity in a clinically relevant context, we evaluated susceptibility across representative ESKAPEE pathogens alongside Rif (Table 2). CorA displayed intrinsic activity (without PMBN) only against *A. baumannii* and the Gram-positive organisms *S. aureus* ATCC 25923 (MIC 0.5 µg/mL) and *E. faecalis* OG1X (MIC 8 µg/mL). All Gram-negative pathogens tested—*P. aeruginosa* PA14, *K. pneumoniae* AZ99, *E. cloacae* ATCC 23355, and *E. coli* MC4100—required PMBN co-treatment to reveal CorA activity, with post-permeabilization MICs ranging from 2 to 8 µg/mL. Notably, *P. aeruginosa* retained high-level resistance to Rif (>128 µg/mL) even with PMBN, suggesting additional intrinsic resistance mechanisms beyond outer membrane impermeability. In all organisms tested, Rif exhibited equal or greater potency than CorA under comparable conditions.

### 2.4. Lack of Cross-Resistance Between CorA and Rif

Given that CorA and Rif target distinct sites within RNAP, we assessed whether resistance to one compound confers cross-resistance to the other (Table 2). *A. baumannii* ATCC 17978.EM3 carrying Rif resistance-conferring *rpoB* substitutions H535Y or S540Y, which conferred >128-fold increases in Rif MIC, retained full susceptibility to CorA (MICs of 1–2 µg/mL, comparable to the parent strain). A partial Rif resistance mutation, *rpoB-S521F*, similarly did not reduce CorA susceptibility. Conversely, the CorA-resistant *A. baumannii rpoB-L1345F* allele did not alter Rif susceptibility. Analogously, a Rif-resistant derivative of *K. pneumoniae* AZ99 with MIC > 128 µg/mL Rif retained susceptibility to CorA (MIC > 128 µg/mL without PMBN, but 4 µg/mL with PMBN), comparable to the Rif-susceptible parent strain. These findings confirm the complete absence of cross-resistance between Rif and CorA, consistent with their non-overlapping binding sites within RNAP.

### 2.5. CorA and Rif Exhibit No Pharmacological Interaction

Given the mechanistic independence of CorA and Rif, and the theoretical appeal of combining two RNAP inhibitors targeting distinct sites, we evaluated their interaction using fractional inhibitory concentration (FIC) index assays (Table 3). Checkerboard microdilution experiments across a matrix of CorA and Rif concentration combinations yielded FIC index values = 1, consistent with indifference (no interaction), similar to FIC index values for CorA or Rif in combination with the control antibiotic ciprofloxacin. These data indicate that simultaneous exposure to both compounds at clinically relevant concentration ratios neither enhances nor impairs activity relative to each compound alone.

### 2.6. Serum Inactivation Reduces CorA In Vitro Activity

To assess the impact of serum on CorA activity, we evaluated MICs under physiologically relevant serum conditions (Table 4). In 50% human serum/50% M9 minimal media, CorA MICs increased dramatically to ≥128 µg/mL for all strains tested, including *A. baumannii* ATCC 17978, AB5075, and the efflux-deficient 17978.EM3 strain, as well as *S. aureus* ATCC 25923 and *E. faecalis* OG1X. Critically, co-treatment with PMBN failed to restore CorA activity under serum conditions for *A. baumannii*, in contrast to the robust potentiation observed in Mueller-Hinton broth (MHB). The serum-mediated loss of activity in *S. aureus* and *E. faecalis* is also notable because membrane permeability does not seem to be a barrier for these species, indicating that the effect cannot be attributed to altered membrane penetration. Taken together, this profile is consistent with sequestration of CorA by serum proteins or lipoproteins. Indeed, serum binding of CorA has been documented at up to 98% across multiple species [21], and the loss of activity observed in 50% human serum likely reflects the greatly reduced free-compound fraction at the concentrations tested rather than intrinsic loss of potency. Rif, by contrast, retained activity in 50% serum across all species, with MICs equivalent to or lower than standard MHB conditions, confirming that the CorA serum effect is compound-specific rather than a general property of the assay format. The translational significance of these findings is, however, tempered by demonstrated in vivo efficacy across multiple animal models and against a diverse range of bacterial pathogens.

### 2.7. CorA Lacks Efficacy in the Galleria mellonella Larval Infection Model

**Figure 2:**
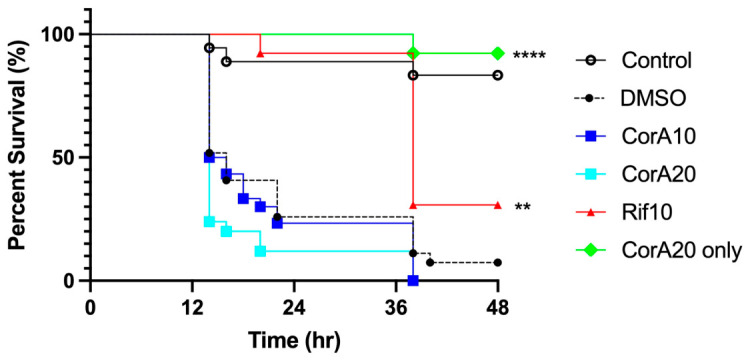
*G. mellonella* larval survival following lethal *A. baumannii* AB5075(O) infection and antibiotic treatment. Larvae were infected with a lethal inoculum and treated 1 h post-infection with DMSO vehicle (filled black circles), 10 mg/kg CorA (blue squares; CorA10), 20 mg/kg CorA (cyan squares; CorA20), or 10 mg/kg rifampin (red triangles; Rif10). Uninfected larvae receiving 20 mg/kg CorA (CorA20 only; green diamonds) served as compound toxicity controls. Uninfected control in open black circles. Survival was monitored every 12 h for 48 h. n = 13–30 per group. ** *p* < 0.01, **** *p* < 0.0001 versus infected + DMSO group by log-rank test.

We evaluated CorA in the *G. mellonella* larval infection model to assess in vivo activity (Figure 2). Larvae infected with *A. baumannii* AB5075 and treated with CorA at 10 or 20 mg/kg succumbed to infection at rates indistinguishable from DMSO vehicle controls (*p* > 0.05), while Rif at 10 mg/kg provided significant protection (*p* < 0.01). Compound toxicity was excluded, as larvae receiving 20 mg/kg CorA without infection survived throughout the 48 h observation period; notably, 100 mg/kg CorA was toxic to larvae, precluding evaluation at doses comparable to those used in other animal models. These data demonstrate that CorA does not provide protection in this model under the conditions tested.

### 2.8. CorA Reduces Established A. baumannii Biofilms

**Figure 3:**
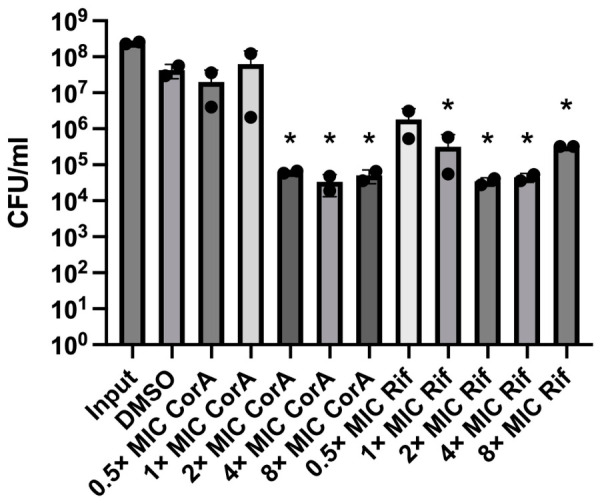
Eradication of established *A. baumannii* AB5075(O) biofilms by CorA and Rif across a range of concentrations relative to planktonic MIC. Mature 24 h biofilms were treated with 0.5×, 1×, 2×, 4×, or 8× MIC CorA or rifampin (Rif) for 24 h. Input (pre-treatment) and DMSO vehicle controls are shown. Viable CFU/mL were enumerated by serial dilution plating. Average of two biological replicates performed in technical duplicate. * *p* < 0.05 compared to DMSO-treated biofilm.

Next, we investigated whether CorA retains activity against *A. baumannii* in the biofilm state. Mature 24 h AB5075 biofilms were treated with a range of CorA concentrations (0.5× through 8× planktonic MIC) and viable colony-forming units (CFU) were quantified after 24 h (Figure 3). Sub-MIC concentrations (0.5× and 1× MIC CorA) did not significantly reduce biofilm CFU compared to DMSO controls. However, treatment at ≥4× MIC resulted in a statistically significant reduction of ~3–4 log CFU/mL compared to DMSO-treated biofilms (*p* < 0.05). Rif treatment at equivalent multiples of its planktonic MIC produced comparable biofilm reduction at ≥1× MIC, with significant reductions also observed at ≥2× MIC. These data indicate that CorA possesses activity against established biofilms at concentrations achievable in plasma and tissues such as lung and bone [19], and that both CorA and Rif can reduce, but not fully eradicate, mature *A. baumannii* biofilms at such concentrations.

## 3. Discussion

From this study, we provide a comprehensive characterization of CorA activity across the ESKAPEE pathogen panel, benchmarked against Rif, and evaluate its translational potential through resistance, cross-resistance, serum activity, in vivo infection, and biofilm studies. Among the ESKAPEE Gram-negative organisms tested, only *A. baumannii* showed intrinsic CorA susceptibility, whereas outer membrane permeabilization with PMBN or colistin revealed CorA activity against *P. aeruginosa*, *K. pneumoniae*, *E. cloacae*, and *E. coli*, demonstrating that the RNAP target is present and accessible once permeability barriers are overcome. By contrast, Rif demonstrated activity against most Gram-negative organisms tested even without PMBN, reflecting its established capacity to penetrate the Gram-negative outer membrane more efficiently than CorA. The intrinsic activity of CorA against Gram-positive organisms *S. aureus* and *E. faecalis* is consistent with prior reports [12,25]. Collectively, these data confirm that outer membrane impermeability, rather than target conservation, is the dominant barrier to CorA activity in Gram-negative ESKAPEE pathogens examined here.

A key finding of this study is the complete absence of cross-resistance between CorA and Rif, consistent with their non-overlapping binding sites within RNAP. Rif binds within the β-subunit RDDR at the RNA exit channel, while CorA engages the structurally distinct switch region [7,8]. Consequently, *rpoB* mutations that confer high-level Rif resistance in the RDDR (H535Y, S540Y) do not reduce CorA susceptibility, and the CorA resistance-conferring mutation L1345F within the switch region does not affect Rif susceptibility. This reciprocal independence was also observed in a Rif-resistant *K. pneumoniae* clinical isolate, extending the cross-resistance analysis beyond *A. baumannii*. The absence of pre-existing cross-resistance in the Rif-resistant species examined here is an important consideration for clinical development: it means that populations harboring Rif resistance would not have a pre-existing advantage against CorA, and vice versa. This profile is favorable for both combination RNAP-inhibitor strategies and for salvage therapy in Rif-resistant infections. The resistance frequency for CorA (~4.5 × 10^−9^) is consistent with data from other organisms and with previous reports suggesting that CorA selects for resistance less readily than Rif under comparable conditions [22]. Importantly, FIC index analysis revealed pharmacological indifference between CorA and Rif. While the lack of synergy is mechanistically consistent with each inhibitor independently occupying its respective binding pocket within RNAP, whether higher-order synergy might emerge under conditions of partial RNAP occupancy or in specific pharmacodynamic contexts remains an open question that warrants further investigation. It should be noted that Rif, while demonstrating reliable activity against *A. baumannii* in vitro, has shown inconsistent results in clinical trials for *A. baumannii* infections; in particular, rapid emergence of Rif resistance during monotherapy and during combination regimens has been documented, limiting its clinical utility. Our data suggests clinical potential for a triple combination of CorA, Rif, and low-dose colistin might achieve potentiated activity against CRAB while substantially constraining the emergence of resistance to either RNAP-targeting component; such a strategy would merit formal evaluation in future studies.

Although CorA MICs increased to ≥128 µg/mL in 50% human serum for all strains tested, the translational significance of this in vitro finding is tempered by substantial in vivo evidence of CorA efficacy across multiple infection models despite its known high serum binding [25]. CorA has demonstrated activity against *Orientia tsutsugamushi, Chlamydia* spp., *Wolbachia* of filarial nematodes, and *S. aureus* in mammalian hosts at therapeutically relevant doses [13,15,17,19,20,21]. Further, pharmacokinetic studies have shown that CorA accumulates in tissues such as the lung and bone at concentrations exceeding plasma levels [19], suggesting that serum protein binding may facilitate distribution to compartments where target pathogens reside rather than simply limiting bioavailability. One hypothesis is that serum binding protects CorA from isomerization to less active conformational forms during systemic circulation, effectively serving as a reservoir that releases active compound at tissue sites. Rif, a known albumin binder, retained MIC activity across all species in 50% serum, and the differential behavior of the two compounds likely reflects differences in binding affinity, dissociation kinetics, or free-drug fraction at the concentrations tested rather than a fundamental advantage of one scaffold over the other. Taken together, the serum binding profile of CorA warrants consideration in the design of in vitro susceptibility assays but should not be interpreted as a definitive barrier to in vivo utility.

In the *G. mellonella* larval infection model, Rif provided statistically significant protection from lethal *A. baumannii* AB5075 infection at 10 mg/kg, while CorA at doses up to 20 mg/kg provided no detectable benefit; higher doses could not be evaluated, as 100 mg/kg was found to be toxic to larvae. These results indicate that CorA lacks sufficient activity against *A. baumannii* in this model under the conditions tested, though the mechanistic basis remains incompletely resolved. *G. mellonella* larvae possess a large fat body relative to total body volume, and given that CorA is highly lipophilic, sequestration into this compartment may reduce free-compound exposure in hemolymph—analogous to the greater doses required in Mongolian gerbils compared to mice, where higher adiposity similarly alters effective drug distribution [26]. An additional mechanism may involve the insect hemolymph lipid transport system: *G. mellonella* employs lipophorins and apolipophorins as a lipid shuttle, and lipophilic non-self compounds such as microbial toxins and lipopolysaccharides can be sequestered within this system as part of a detoxification response. CorA’s extreme lipophilicity may render it a substrate for this shuttle, effectively reducing the free fraction available for antimicrobial activity and further complicating the pharmacokinetic interpretation of results in this insect model. These outcomes illustrate the complexity of predicting tissue-level exposure and efficacy from blood pharmacokinetics alone, particularly for lipophilic compounds in non-mammalian models. Oral formulations with high bioavailability have been developed and demonstrated in vivo efficacy against *S. aureus* [20], *Orientia tsutsugamushi* and *Rickettsia typhi* in mice [13], and *Wolbachia* in mice, gerbils [15,21] and dogs (manuscript in preparation), establishing a viable path toward clinical development that does not depend on resolving serum binding as a prerequisite.

CorA also demonstrated significant activity against established *A. baumannii* biofilms in vitro. Mature 24 h biofilms treated at ≥4× planktonic MIC exhibited 3–4 log CFU/mL reductions compared to vehicle controls, a magnitude of activity that is notable given the well-documented tolerance of biofilms to antibiotics, often requiring 10–100× planktonic MICs for equivalent killing [27]. The biofilm-active concentrations may be achievable through topical or local delivery strategies such as wound care, catheter-lock solutions, or device coatings, where systemic exposure and serum binding are less relevant. Notably, the antibiofilm potential of CorA extends beyond *A. baumannii*. A recent study by De Benedetti et al. demonstrated that CorA eradicated established *S. aureus* biofilms in vitro across a broad panel of strains—outperforming both Rif and dalbavancin in confocal viability assays—and achieved >4-log_10_ reductions in bacterial load on implanted devices in a murine foreign body infection model, with efficacy comparable to Rif [17]. Given the pharmacological indifference observed between CorA and Rif in planktonic assays and the mechanistically distinct binding sites of these two RNAP inhibitors, evaluation of their combined activity against established biofilms represents a productive future direction. Together with the *A. baumannii* biofilm data presented here, these findings support CorA as a broad-spectrum antibiofilm agent with in vivo proof-of-concept in a relevant device-associated infection context.

Overall, these findings contribute to a growing body of evidence positioning CorA as a mechanistically compelling RNAP inhibitor with a well-characterized activity spectrum. Its ability to overcome Rif resistance, combined with biofilm activity against MDR *A. baumannii*, suggests potential utility as a topically delivered agent in wound or device-associated infection contexts, as well as a component of combination strategies in Rif-resistant settings. While serum binding warrants consideration for systemic applications against ESKAPEE pathogens, the established in vivo efficacy of CorA against *S. aureus*, *Orientia tsutsugamushi*, *Chlamydia* spp., and *Wolbachia* in mammalian models demonstrates that high serum binding does not preclude therapeutic activity, and oral formulations achieving efficacy in multiple species have already been developed. Our data underscores CorA as a mechanistically compelling RNAP inhibitor with demonstrated translational potential, while identifying serum binding and organism spectrum as key parameters to consider in the context of ESKAPEE pathogens specifically. Ongoing work will further define the concentration-effect relationships needed to guide formulation optimization and inform structure-activity relationship studies aimed at maximizing free-drug exposure while preserving RNAP target engagement.

## 4. Materials and Methods

### 4.1. Bacterial Strains and Chemicals

Bacterial strains used in this study are listed in Table S1. *Acinetobacter baumannii* ATCC 17978, utilized for all genetic studies, is a well-characterized antibiotic-susceptible strain isolated from a neonatal meningitis infection. AB5075, MU1984, and PR322 are carbapenem-resistant *A. baumannii* (CRAB) clinical isolates. All pathogens were cultured in Luria–Bertani broth (LB; BD Difco Cat no. 244620, Franklin Lakes, NJ, USA) with antibiotics if appropriate. Research-grade CorA (>91% pure, batch R778) was produced using the heterologous producer strain, *Myxococcus xanthus* DK1622::*pDPO-mxn116-Pvan-Tpase*, as previously described [25]. Stock CorA solutions were prepared by dissolving in 50% dimethyl sulfoxide (DMSO; Sigma-Aldrich Cat no. D4540, St. Louis, MO, USA) and stored at −80 °C until used. Rifampin (Rif; Cat no. PHR1806), ciprofloxacin (Cipro; Cat no. PHR1167) and polymyxin B nonapeptide (PMBN; Cat no. P2076) were obtained from Sigma-Aldrich (St. Louis, MO, USA).

### 4.2. Antimicrobial Susceptibility Testing

Minimal inhibitory concentrations (MICs) were determined using a two-fold microtiter broth dilution method following Clinical and Laboratory Standards Institute (CLSI) standards [28]. Briefly, 10 µL of an exponential phase culture diluted to ~3 × 10^7^ CFU/mL was added to individual wells of a 96-well flat bottom plate (Corning Cat no. 3370, Corning, NY, USA) containing 88 µL Mueller Hinton broth (MHB; BD Difco Cat no. 275730, Franklin Lakes, NJ, USA) or 50% human serum (MP Biomedicals Cat no. 0929301, Santa Ana, CA, USA)/50% M9 minimal media (BD Difco Cat no. 248510), and 2 µL of the antimicrobial compound resuspended in 50% DMSO (ranging from 0 to 256 µg/mL final concentration). MHB mixtures were incubated at 37 °C for up to 20 h and optical density at 600 nm (OD_600_) was read using a Thermo Scientific Spectronic 200 spectrophotometer (Waltham, MA, USA). The MIC was defined as the lowest concentration of compound with an OD_600_ reading equivalent to the no-bacteria control. Serum/M9 mixtures were incubated at 37 °C for up to 48 h and MIC was determined by serial diluting and plating wells for CFU. Serum/M9 MIC assays utilized CFU enumeration rather than OD_600_ turbidity because serum components produce significant baseline absorbance that precludes reliable optical density-based growth determination; longer incubation (48 h) was required to accommodate reduced growth rate in the serum/M9 medium. The MIC was defined as the lowest concentration of compound with CFU ≤ 10^6^ CFU/mL. All MICs were performed in at least triplicate and the median MIC was reported. For MICs performed with PMBN or colistin, 50 µg/mL PMBN or 0.25×–0.5× MIC colistin was added to the medium.

### 4.3. Fractional Inhibitory Concentration (FIC) Index Assays

Interactions between CorA, Rif, and the control antibiotic (ciprofloxacin) were evaluated using a standard checkerboard microdilution assay. Two-fold serial dilutions of each compound were prepared independently and combined in a 96-well flat-bottom plate to generate an 8 × 8 concentration matrix, with each compound ranging from 0 to 4× its MIC against *A. baumannii* ATCC 17978 and AB5075. Plates were inoculated as described for MIC assays and incubated at 37 °C for up to 20 h. The FIC index (FICI) was calculated as (MIC_Drug A_ combo/MIC_Drug A_ alone) + (MIC_Drug B_ combo/MIC_Drug B_ alone). Interactions were interpreted as: synergy, FICI ≤ 0.5; indifference, 0.5 < FICI ≤ 4; antagonism, FICI > 4. Assays were performed in biological duplicate and in technical duplicate, with median MICs reported and utilized for FIC calculations.

### 4.4. Biofilm Elimination Studies

10^5^ CFU AB5075 (O) was seeded into 200 µL MHB media in flat-bottom 96-well plates and incubated at 37 °C for 24 h to establish biofilms. Biofilms were washed twice with 200 µL phosphate-buffered saline (PBS, pH 7.2; BD Difco Cat no. 223142) then treated with DMSO, or 0.5×, 1×, 2×, 4×, or 8× MIC CorA or Rif (based on AB5075 (O) planktonic MICs) ± 50 µg/mL PMBN resuspended in 200 µL MHB for 24 h at 37 °C. Biofilms were washed once with 200 µL PBS, fully resuspended in 100 µL PBS, and serial diluted to enumerate CFU by plating on LB agar.

### 4.5. Galleria mellonella Infection Model

*G. mellonella* larvae (200–250 mg) were purchased from Premium Crickets (Winder, GA, USA) and maintained at room temperature until use. Larvae were injected with a lethal inoculum of *A. baumannii* AB5075 (~5 × 10^5^ CFU/larva) into the last left proleg. Antibiotic treatment (DMSO vehicle, 10 or 20 mg/kg CorA, or 10 mg/kg Rif prepared in 2 µL volume in 50% DMSO) was administered 1 h post-infection via injection into the right proleg. Uninfected larvae receiving 20 mg/kg CorA served as compound toxicity controls. Survival was monitored repeatedly over 48 h; larvae were scored as dead upon failure to respond to touch. Statistical significance was determined by log-rank (Mantel–Cox) test. Group sizes ranged from 13 to 30 larvae, reflecting the availability of appropriately sized larvae at the time of the experiment; all groups were of sufficient size to detect the effect sizes observed by log-rank analysis.

### 4.6. Generation of CorA and Rif Resistant Mutants and Whole-Genome Sequencing

ATCC 17978.EM3 was grown to an OD_600_ = 0.8 in 2 mL LB. Cells were washed twice in fresh LB, resuspended in 200 μL, then plated on LB agar containing 4 µg/mL CorA or Rif (4× MIC) and grown at 37 °C for 48 h. Suppressor mutation frequency was calculated as CFU on selective agar divided by total CFU plated. Genomic DNA from four independent suppressor mutants generated from a single selection experiment and the corresponding input strain was isolated and sent to SeqCenter LLC (Pittsburgh, PA, USA) for library preparation, sequencing on the NextSeq 2000 platform at 2 × 150 bp depth, and alignment/variant calling to ATCC17978-mmf (GenBank Accession: CP012004.1).

### 4.7. PCR Amplification of rpoB-L1345F and Allelic Exchange

Allelic exchange was facilitated using a RecET-mediated recombineering system as described [29]. The region flanking the CorA-resistant *rpoB* allele was amplified using primers RpoB* F (5′-CCATTTGGTCACCGTCGAAGTC) and RpoB* R (5′-GCACGTGATACTAAGTTAGGTGC) using a high-fidelity DNA polymerase (Phusion Hot Start II, Thermo Fisher Scientific, Waltham, MA, USA) under the following thermocycling conditions: 98 °C for 3 min initial denaturation; 35 cycles of 98 °C for 30 s, 60 °C for 30 s, 72 °C for 3 min; and final extension at 72 °C for 5 min, performed on a Eppendorf Vapo.Protect Mastercycler Pro thermocycler (Eppendorf, Hamburg, Germany). A purified PCR product was used for electroporation of ATCC 17978 carrying IPTG-inducible RecET expression. Transformants were selected on LB agar containing 32 µg/mL CorA and confirmed by sequencing using primer RpoB* seq (5′-CCATCTGAGTCCGTTTTCTTACGC).

## 5. Conclusions

This study provides a comprehensive characterization of corallopyronin A activity across the ESKAPEE pathogen panel and evaluates its translational potential. CorA exhibits intrinsic activity against *A. baumannii*, including carbapenem-resistant clinical isolates, and against Gram-positive ESKAPEE organisms, while activity against other Gram-negative pathogens requires outer membrane permeabilization. The absence of cross-resistance between CorA and rifampin is confirmed at both the biochemical and genetic levels in *A. baumannii* and *K. pneumoniae*, supporting their combined use as a dual RNAP-inhibitor strategy. High serum binding reduces CorA activity in vitro but does not preclude in vivo efficacy based on established data across multiple animal models and pathogens. CorA demonstrates significant antibiofilm activity against established *A. baumannii* biofilms at supratherapeutic concentrations. Together, these findings define both the opportunities and the translational boundaries for CorA as an RNAP-targeting therapeutic candidate against ESKAPEE priority pathogens.

## 6. Patents

AH and KP hold patents for CorA to treat human and animal filarial infections (US 9168244 B2, US 9687470 B2, EP 2704708 B1) and for solid oral formulations (EP 4142696).

## Figures and Tables

**Table 1:** CorA MICs of *A. baumannii* isolates with various efflux pump mutations or permeabilization agents.

Strain (+Condition)	CorA MIC (µg/mL)
ATCC 17978	16
+50 µg/mL PMBN	0.5
+0.25× MIC Colistin	1
+0.5× MIC Colistin	<0.0625
ATCC 17978 Δ*adeB*	16
ATCC 17978 *adeG::kan*	16
ATCC 17978 Δ*adeJ*	1
ATCC 17978.EM3 (Δ*adeB* Δ*adeG* Δ*adeJ* Δ*surA*)	1
ATCC 17978 *adeL::kan (adeFGH+)*	16
ATCC 17978 *adeN::kan (adeIJK+)*	32
ATCC 17978 *rpoBL1345F*	64
AB5075 (O)	32
+50 µg/mL PMBN	0.5
+0.25× MIC Colistin	4
+0.5× MIC Colistin	1
AB5075 Δ*adeJ* (O)	4
MU1984	32
+50 µg/mL PMBN	1
MU1984 Δ*adeJ*	8
PR322	16
+50 µg/mL PMBN	2
PR322 Δ*adeJ*	4

Colistin MIC = 1 µg/mL; PMBN = polymyxin B nonapeptide.

**Table 2:** CorA and Rif MICs ± 50 µg/mL PMBN of ESKAPEE pathogens with and without Rif resistance-conferring *rpoB* mutations.

Strain	CorA	CorA + PMBN	Rif	Rif + PMBN
*A. baumannii* ATCC 17978	16	0.5	4	<0.25
*A. baumannii* ATCC 17978 *rpoB-L1345F*	64	4	4	<0.25
*A. baumannii* AB5075 (O)	32	1	2	<0.25
*A. baumannii* MU1984	32	1	2	<0.25
*A. baumannii* PR322	16	2	2	<0.25
*P. aeruginosa* PA14	>128	<0.25	>128	<0.25
*K. pneumoniae* AZ99	>128	4	32	<0.25
*K. pneumoniae* AZ99 Rif resistant	>128	4	>128	>128
*S. aureus* ATCC 25923	0.5	0.5	<0.25	<0.25
*E. faecalis* OG1X	8	8	4	4
*E. cloacae* ATCC 23355	>128	2	8	<0.25
*E. coli* MC4100	>128	8	8	<0.25
*A. baumannii* 17978.EM3	1	—	1	—
*A. baumannii* 17978.EM3 *rpoB-H535Y*	1	—	>128	—
*A. baumannii* 17978.EM3 *rpoB-S540Y*	2	—	>128	—
*A. baumannii* 17978.EM3 *rpoB-S521F*	2	—	16	—

All MICs in µg/mL. PMBN = polymyxin B nonapeptide. Rif = rifampin. —, not tested.

**Table 3:** CorA and Rif fractional inhibitory concentration (FIC) index values.

Strain–Drug A + Drug B	MIC_Drug A_ Alone	MIC_Drug A_ Combo	MIC_Drug B_ Alone	MIC_Drug B_ Combo	FIC
*A. baumannii* ATCC 17978–CorA + Rif	16	8	4	2	1
*A. baumannii* AB5075 (O)–CorA + Rif	32	2	16	1	1
*A. baumannii* 17978–CorA + Cipro	16	16	0.25	0.25	2
*A. baumannii* 17978–Rif + Cipro	4	4	0.25	0.25	2

All MICs in µg/mL. Rif = rifampin. Cipro = ciprofloxacin.

**Table 4:** CorA and Rif MICs ± 50 µg/mL PMBN of select ESKAPEE pathogens in 50% human serum/50% M9 minimal media.

Strain	CorA	CorA + PMBN	Rif
*A. baumannii* ATCC 17978	128	128	4
*A. baumannii* AB5075 (O)	128	128	2
*A. baumannii* 17978.EM3	128	—	1
*P. aeruginosa* PA14	>128	—	>128
*K. pneumoniae* AZ99	>128	—	2
*S. aureus* ATCC 25923	>128	—	2
*E. faecalis* OG1X	>128	—	4

All MICs in µg/mL. Rif = rifampin. —, not tested.

## Data Availability

The original contributions presented in this study are included in the article/Supplementary Material. Further inquiries can be directed to the corresponding author.

## Supplementary Materials

The following supporting information can be downloaded at: https://www.mdpi.com/article/10.3390/antibiotics15070665/s1, Table S1: Bacterial strains used in this study. References [30,31,32,33,34,35,36,37,38] are cited in the supplementary materials.
