# Supplementary material for: Activity of Corallopyronin A Against ESKAPEE Pathogens: Potential and Translational Implications
Source: Antibiotics (Basel). 2026 Jul 8;15(7):665. doi: 10.3390/antibiotics15070665 (PMC13405735; doi:10.3390/antibiotics15070665)
Supplement: Supplementary file 1 [file antibiotics-15-00665-s001.zip › antibiotics-4404820-supplementary.pdf]

Supplementary Data.

Table S1. Bacterial strains used in this study.

Strain	Genotype / Description	Source or Reference
Acinetobacter baumannii		
ATCC 17978	Wild-type; antibiotic-susceptible reference strain; originally isolated from neonatal meningitis cerebrospinal fluid. Complete genome sequenced (GenBank CP012004.1).	[30]
ATCC 17978 *ΔadeB*	ATCC 17978 carrying an in-frame deletion of *adeB* (AdeABC efflux pump membrane fusion protein component); AdeABC-null.	This study
ATCC 17978 *adeG::kan*	ATCC 17978 with kanamycin-cassette insertional disruption of *adeG* (AdeFGH efflux pump membrane fusion protein component); AdeFGH-null.	This study
ATCC 17978 *ΔadeJ*	ATCC 17978 carrying an in-frame deletion of *adeJ* (AdeIJK efflux pump membrane fusion protein component); AdeIJK-null.	This study
ATCC 17978.EM3	ATCC 17978 with triple efflux pump deletions (*ΔadeB ΔadeG ΔadeJ*) and deletion in *surA* (periplasmic chaperone). Used as permeable, low-efflux background for spontaneous resistance selection.	[31]
ATCC 17978 *adeL::kan*	ATCC 17978 with kanamycin-cassette disruption of *adeL* (transcriptional repressor of AdeFGH); results in constitutive AdeFGH overexpression.	This study
ATCC 17978 *adeN::kan*	ATCC 17978 with kanamycin-cassette disruption of *adeN* (transcriptional repressor of AdeIJK); results in constitutive AdeIJK overexpression.	This study
ATCC 17978 *rpoB-L1345F*	ATCC 17978 carrying engineered CorA resistance-conferring *rpoB* substitution L1345F (switch region).	This study
ATCC 17978.EM3 *rpoB-H535Y*	ATCC 17978.EM3 background with rifampin resistance-conferring *rpoB* substitution H535Y.	This study
ATCC 17978.EM3 *rpoB-S540Y*	ATCC 17978.EM3 background with rifampin resistance-conferring *rpoB* substitution S540Y.	This study
ATCC 17978.EM3 *rpoB-S521F*	ATCC 17978.EM3 background with rifampin resistance-conferring *rpoB* substitution S521F.	This study
AB5075 (O)	Carbapenem-resistant clinical isolate recovered from osteomyelitis infection at Walter Reed Army Medical Center, USA in 2008 (Genbank CP008706.1).	[32]
AB5075 *ΔadeJ* (O)	AB5075 (O) carrying an in-frame deletion of *adeJ;* AdeIJK-null.	This study
MU1984	Carbapenem-resistant clinical isolate recovered from blood infection in Atlanta, Georgia, USA in 2012 (Genbank CP096818.1).	Provided by D. Weiss (Emory University)
MU1984 *ΔadeJ*	MU1984 carrying an in-frame deletion of *adeJ;* AdeIJK-null.	This study

PR322	Carbapenem-resistant clinical isolate from Antibacterial Resistance Leadership Group (ARLG-1820).	Provided by R. Bonomo (Case Western Reserve University / Louis Stokes VA Medical Center)
PR322 *ΔadeJ*	PR322 carrying an in-frame deletion of *adeJ;* AdeIJK-null.	This study
Pseudomonas aeruginosa		
PA14	Wild-type, highly virulent human clinical burn wound isolate collected in Pennsylvania, USA.	[33]
Klebsiella pneumoniae		
AZ99	Mouse-passage streptomycin-resistant derivative of AZ10 (stool isolate; ST 1322; *wzi* 372).	[34]; Provided by A. Zafar (Emory University)
AZ99 Rif^R	Rifampin-resistant derivative of AZ99.	Provided by A. Zafar (Emory University)
Staphylococcus aureus		
ATCC 25923	Methicillin-susceptible reference strain.	[35]
Enterococcus faecalis		
OG1X	Plasmid-free, streptomycin- and fusidic acid-resistant derivative of OG1; widely used laboratory reference strain.	[36]
Enterobacter cloacae		
ATCC 23355	Standard reference strain.	[37]
Escherichia coli		
MC4100	*F^- araD139 Δ(argF-lac)U169 rpsL150(Str^R) relA1 flbB5301 deoC1 ptsF25 rbsR*; standard K-12 laboratory derivative.	[38]; Provided by M. Grabowicz (Emory Univerisity)

Abbreviations: ATCC = American Type Culture Collection.
